# Mechanisms through which sleep influences intrusive memories: protocol for a trauma film paradigm study

**DOI:** 10.1093/sleepadvances/zpag022

**Published:** 2026-02-14

**Authors:** Jessica Ogden, Laura Jobson, Sean P A Drummond

**Affiliations:** School of Psychological Sciences, Monash University, Clayton, VIC, Australia; Turner Institute for Brain and Mental Health, Monash University, Clayton, VIC, Australia; School of Psychological Sciences, Monash University, Clayton, VIC, Australia; Turner Institute for Brain and Mental Health, Monash University, Clayton, VIC, Australia; School of Psychological Sciences, Monash University, Clayton, VIC, Australia; Turner Institute for Brain and Mental Health, Monash University, Clayton, VIC, Australia

**Keywords:** sleep/wake cognition, REM sleep, posttraumatic stress disorder, learning and memory

## Abstract

Intrusive memories are common following trauma exposure. However, they can develop into a distressing symptom of posttraumatic stress disorder (PTSD). Recent research examining the role of sleep in the development of intrusive memories demonstrates overnight sleep or a daytime nap following analog trauma exposure (ie, a trauma film), compared to a similar period of wake, results in fewer intrusive memories in the following week. This is consistent with research examining participants who have recently experienced trauma, demonstrating sleep disturbances are highly associated with the development of intrusive memories. However, the mechanisms underlying this are not well understood. Identifying those mechanisms is critical to improving interventions to reduce intrusive memories, and thus, the impacts of posttraumatic stress disorder. This study aims to investigate two potential mechanisms underlying the relationship between sleep and intrusive memories; namely, whether sleep-related memory consolidation and/or executive control over spontaneous cognition explains this relationship. These aims will be investigated through an experimental trauma film study in a healthy adult population (*n* = 114), where we will compare the effects of a 2-h nap versus 2-h controlled waking period on intrusive memories for the trauma film. In the subsequent week, we will examine the relationship between sleep the previous night and intrusive memories the following day. The outcomes of this project will provide important insights regarding the mechanism(s) explaining the relationship between sleep and intrusive memory frequency. Findings will provide further direction for research investigating sleep-related effects on intrusive memories and may have implications for intervention post trauma exposure.

**Statement of Significance**

Sleep following experimental trauma exposure reduces the frequency of intrusive memories. In trauma-exposed populations, sleep is also found to play a role in the development and maintenance of posttraumatic stress disorder symptoms, including intrusive memories. However, there is limited understanding as to how sleep exerts this influence. This study is attempting to fill this gap in the literature by investigating whether memory consolidation for trauma stimuli and/or executive control over spontaneous cognition mediates the relationship between sleep and intrusive memories. Findings from this study will provide insights into sleep’s effect on intrusive memories and may identify new targets for intervention following trauma exposure.

## Introduction

Intrusive memories are spontaneous, involuntary intrusions of memories, typically sensory experiences (eg, sights, sounds, smells), into conscious awareness [1–3]. While in many individuals intrusive memories tend to dissipate over time following trauma, for those with posttraumatic stress disorder (PTSD), this symptom can remain distressing and persistent for many years [1]. Sleep disturbance, another common symptom following trauma, is conceptualized as both a symptom of PTSD and a comorbid disorder [4]. Evidence suggests sleep disturbance prior to [5–7] and early after [7–9] trauma predicts the development of PTSD. Sleep disturbance is also related to intrusive memories specifically. For example, sleep disturbance is associated with more frequent intrusive memories in the week after experiencing trauma [10, 11]. Even many years after trauma exposure, disturbed sleep the previous night is still associated with more intrusive memories the following day [12].

Experimentally, the effects of sleep on intrusive memories have been established through the utilization of the trauma film paradigm [13–16]. The trauma film paradigm has been used as an analog of trauma and is a well-established paradigm within the PTSD field, specifically for studying intrusive memories [17]. It is shown to reliably elicit intrusive memories and has been utilized to study a range predictors of intrusive memory development [17], including the role of sleep. In these studies, participants view a distressing film and are allocated to either a sleep or a wake condition following the film. Participants then report on film-related intrusive memories and associated distress over several days [18]. Four separate meta-analyses have each concluded a period of intact sleep following exposure to the analog trauma reduces the number of intrusive memories experienced [19–22]. This has been found in studies examining sleep compared to total overnight sleep deprivation [13], partial sleep deprivation [23], wake during the day [16], or a nap compared to the same period of wake [14, 15]. Several studies also point to a specific role for rapid eye movement (REM) sleep, which, compared to the absence of REM, leads to both fewer intrusions [24, 25] and lower distress associated with intrusions [26]. Overall, both research examining clinical populations and findings of experimental trauma film paradigm studies demonstrate healthy sleep after trauma appears critical for reducing the number of intrusive memories.

However, the mechanism(s) by which sleep reduces intrusive memories remains unknown. Models of intrusive memory development in PTSD propose consolidation of the trauma memory into autobiographical memory networks is one mechanism for reducing intrusive memories [27–29]. This consolidation of the trauma memory is thought to be indicated by enhanced explicit memory, such as an accurate voluntary recall of the trauma memory [16] and improved ability to distinguish between experienced trauma stimuli and similar but new stimuli [13, 30]. As sleep plays an important role in the consolidation of emotional memories [16] and is found to improve voluntary recall of trauma stimuli, yet reduce intrusive memories of trauma stimuli [13], several studies argue the sleep-related reduction in intrusive memories is due to memory consolidation occurring over a period of sleep compared to wake [13, 16, 23, 31].

A second potential mechanism explaining the role of sleep in intrusive memories relates to sleep disturbances increasing disruptive spontaneous cognition (eg, mind wandering), which in turn accounts for the involuntary intrusion of trauma memories into awareness [19, 32]. Mind wandering occurs when, instead of being focused on task relevant demands, attention is captured by internal stream of thought [33]. Notably, increased mind wandering has been observed following both self-reported poor sleep [34] and experimental sleep deprivation [19, 35] and may occur via sleep’s effect on reduced executive control [32]. Therefore, reduced top-down executive control over traumatic autobiographical memories would be expected to increase the likelihood of the memories becoming intrusive. This process may be exacerbated by sleep deprivation, due to the resultant negative impact on executive control [19, 36]. As people frequently experience sleep difficulty following trauma [37], this explanation may represent an important mechanism for the early maintenance of intrusive memories.

In summary, sleep disturbance plays a role in the development of intrusive memories following trauma exposure [19–22]. The critical gap in the literature is understanding the mechanism(s) whereby sleep exerts this influence [36]. The current project will address this through an experimental study in healthy adults. We are utilizing a nap vs wake design, rather than overnight sleep vs sleep deprivation design, as in addition to reducing the frequency of intrusive memories, naps are found to improve memory for a range of emotional [23, 38] and neutral stimuli [39]. Utilizing a nap/wake paradigm also ensures intrusion frequency is due to sleep rather than the effect of total sleep deprivation (eg, if using an overnight wake period) or potential circadian confounds of comparing a day of wake to a night of sleep immediately following exposure to the trauma film. The current study will be the first to examine memory consolidation as a mechanism linking sleep to intrusive memories using a nap versus wake design, as well as the first to examine mind wandering as a mechanism linking sleep to intrusive memories, using any type of design. Additionally, we will examine natural variation in sleep across multiple nights following analog trauma exposure to examine the relationships between sleep the night before and both mind wandering and intrusive memories the following day.

### Aims and hypotheses

The overall objective of this project is to examine two mechanisms underlying the relationship between sleep and the development of involuntary intrusive memories, utilizing a well-established experimental analog trauma exposure. The two mechanisms are:

1. Improved memory consolidation over periods of sleep leads to integration of the trauma memory into the broader memory network, thereby reducing intrusive memories.
2. Sufficient sleep quality/quantity facilitates executive control over spontaneous cognition, including intrusive memories, decreasing the likelihood of their occurrence.

This study will employ a between-subjects design, with two conditions: (1) 2-h daytime nap opportunity, measured using polysomnography; and (2) equal length of controlled waking. Each condition will include *n* = 57 healthy adults, aged 18–49 years old (total *N* = 114, aiming for equal numbers of women and men). OSF preregistration: https://doi.org/10.17605/OSF.IO/MWAHD

Aim 1: Examine the effect of a daytime nap (vs. wake) on intrusive memory frequency.

Hypothesis 1. We will replicate the established finding that a nap, relative to wake, will result in a reduced number of intrusive memories [14, 15] in the 5 days following viewing the trauma film.

Aim 2: Investigate the effect of sleep-related memory consolidation on intrusive memory frequency.

Hypothesis 2a. A nap, relative to wake, will lead to greater recognition memory for the trauma film stimuli immediately after the nap/wake period, as measured by improved recognition of trauma stimuli from the film and reduced endorsement of lure stimuli (film images never seen).

 

Hypothesis 2b. Recognition memory for the trauma film immediately after the nap/wake period will mediate the relationship between sleep condition (nap vs wake) and the number of intrusive memories in the following 5 days.

Aim 3: Investigate the effect of overnight sleep on mind wandering and intrusive memory frequency over a 5-day period following analog trauma exposure.

Hypothesis 3a. Reduced quantity of sleep at night will be associated with increased mind wandering and greater frequency of intrusive memories the next day.

 

Hypothesis 3b. Mind wandering will mediate the relationship between sleep the previous night and intrusive memories the following day.

## Methods and Materials

### Study design

**Figure 1:**
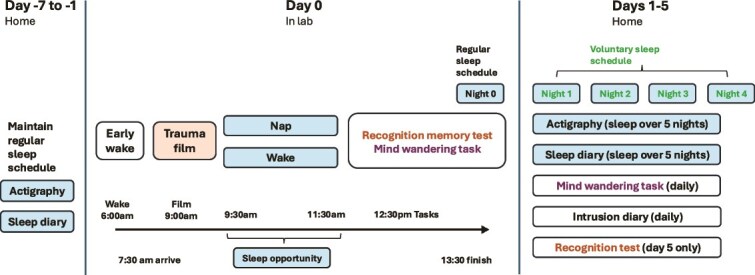
Timeline of study participation. During days −7 to −1, participants maintain a regular sleep schedule. Day 0 (lab session) is indicative of a participant with a habitual wake time of 8 am. Lab session timing depends on participant sleep schedule. All sessions are a maximum of 6 h long. For the night following the lab session, participants follow the regular sleep schedule. For the remaining four nights of sleep, participants sleep ad libitum. During days 1–5, participants complete a daily mind wandering task (sustained attention task), fill out a diary for intrusive memories, and repeat the recognition task on the final day.

There are three phases of participation in the study. Figure 1 outlines the timeline of study participation. During phase 1, 1 week prior to the lab session (days −7 to −1), participants maintain a regular sleep schedule and document adherence with a daily sleep diary and wear actigraphy. On the first day of the study (day −7), participants meet with the researchers to confirm their sleep schedule, learn how to complete a sleep diary, practice a task to be completed during the lab session, and are provided with the actigraphy device.

Phase 2 is the lab session (day 0). Participants wake up 2 h prior to their habitual wake time. This is to ensure sufficient sleep during the nap and to mildly sleep-deprive the wake group. After reporting to the lab in the morning participants are set-up with polysomnography. Following this, they view an analog trauma film in a dark sound attenuated room. Participants are then randomly assigned to one of the two groups: an experimental (nap) group and a controlled (wake) group, for a 2-h period. During this time, those in the wake group can engage in specific activities provided by the researchers including coloring and puzzles. They are unable to use social media, watch videos, view photos, read, or engage in university study/other work during this time. At the end of this experimental period, polysomnography equipment is removed and there is a 30-min delay period for both groups, to allow for the effects of sleep inertia to subside for the nap group. Participants then complete a recognition memory task for images from the trauma film and a sustained attention task to measure mind wandering (hereinafter referred to as “mind wandering task”) in the same sound attenuated room. Following this, participants are provided with instructions for completing an intrusive memory diary.

Phase 3 includes the remaining 5 days of the experiment. Over the next five nights, we will continue to monitor participant sleep with diaries and actigraphy. Participants complete daily intrusive memory diaries, starting as soon as they leave the lab, with six entries at minimum. During each day, they complete the same mind-wandering task completed during the lab session. The recognition task is repeated on day 5. Following study completion, participants attend a debrief meeting.

### Participants and recruitment

#### Sample size

Our target sample size is 114 participants, with 57 in each condition. This study is powered primarily on Hypothesis 1, the group effect (nap vs. wake) on intrusive memory frequency. Relevant effect sizes were derived from a variety of prior studies, which have also been reported within at least one of four meta-analyses. Previous studies [14, 15, 25] examining the effect of a nap compared to wake on intrusion frequency have detected small to medium effect sizes (Hedges’ *g* = 0.38 [26] and 0.69 [15]) for between-group differences in intrusive memory frequency in the days following the experimental manipulation. Gvozdanovic et al. (2023) [14] also found a nap led to significantly fewer intrusive memories than the wake group, however, an effect size was not provided. For studies examining recognition memory for the trauma film following overnight sleep compared to wake or partial sleep deprivation [13, 23, 40], we calculated a sample size weighted average for Cohen’s *d*, finding a medium effect size of 0.5 for between-group differences where sleep, relative to wake, led to improved recognition memory for the film. Consistent with these studies, to detect a medium effect size of 0.5, with a two-tailed α of 0.05 and 80 per cent power, 57 participants are needed per group (114 participants in total) [41].

#### Status and timeline

Data collection began on June 28, 2024, with the first participant commencing the study on July 11, 2024. Data collection will continue until reaching the target sample size, which is estimated to be July 2026. Data will be available after the publication associated with those data.

#### Randomization

During the lab session (day 0), participants are randomly assigned to one of the two groups: an experimental (nap) group and a control (wake) group. All participants are mildly sleep deprived the morning of the lab session to increase the probability individuals in the nap group can both sleep and experience REM sleep. Participants are blinded to group allocation until immediately prior to the nap/wake period. Researchers are blinded to participant group allocation until after polysomnography set-up is complete. Thus, the polysomnography set-up for the wake group serves as a sham and to allow verification of sustained wakefulness during the experimental period. Groups of six participants are block randomized in equal numbers to each group and film order (out of two films), stratified by sex, to increase the likelihood of equal assignment over time. A researcher not involved in the administration of lab sessions (SPAD) created a spreadsheet of participant allocation to group and film order using https://www.sealedenvelope.com/simple-randomiser/v1/lists. Cell blackout is used to conceal allocation until the researcher responsible for project administration (J.O.) has completed polysomnography set-up.

#### Recruitment and inclusion criteria

Participants are from a healthy population, aged between 18 and 49 years. Participants are recruited through advertisements on the Monash University Clayton and Caulfield campuses and word-of-mouth. The study is also advertised through social media.

Participants are screened to determine if they meet eligibility criteria via a phone call and online screening questionnaire. Table 1 outlines exclusion criteria. Table 2 outlines self-report questionnaires used as part of the screening process and further self-report questionnaires administered during the study. Informed consent is obtained following the phone call and prior to completing the screening questionnaire.

**Table 1:** Exclusion Criteria

Category	**Exclusion criteria**
*Psychological and neurodevelopmental disorders*	Current psychiatric conditions: bipolar disorder, psychosis, schizophrenia, depression, panic disorder or OCD, all personality disorders, substance use disorder
	Lifetime diagnosis of PTSD
	Score of 31 and above on the PCL-5 (assessment of current PTSD symptoms) for any Criterion A traumatic event
	Taking anti-depressant or anxiety medication
	Diagnosis of ADHD and/or taking stimulant medication
*Sleep*	Current sleep disorders
	Having a habitual sleep window that falls outside of a 22:00–01:00 bedtime and 06:00–09:00 wake time
	Taking sleep medication
*Neurological conditions*	All neurological conditions (including epilepsy), history of stroke, or acquired brain injury (except for mild concussions involving loss of consciousness <15 min)
	Experiencing frequent migraines, have experienced a migraine in the past 3 months or if migraines are triggered by mild sleep deprivation and/or flashing lights
*Language*	Not fluent in English
	Speaks French (Conversational level). One of the films involves dialogue in the French language. No subtitles are included
*Other*	Color blindness and/or any visual impairment/uncorrected visual acuity
	Experiencing hearing difficulties or ear pain
	Report having watched either of the films

**Table 2:** Self-Report Measures by Timepoint

**Self-report measures by timepoint**	**Description**
*Initial screening questionnaire*	
Epworth Sleepiness Scale [42]	8-item questionnaire measuring daytime sleepiness, cutoff score > 10.
STOP-BANG Questionnaire [43]	8-item questionnaire screening for Obstructive Sleep Apnea. Cutoff score > 3. Participants with scores above cutoff for both the Epworth Sleepiness Scale and STOP-BANG are excluded due to OSA risk.
Pittsburgh Sleep Quality Index (PSQI) [44]	19-item questionnaire which assesses sleep quality and disturbances over the previous month. Cutoff score > 5.
Alcohol Use Disorders Identification Test (AUDIT) [45]	10-item screening tool to assess alcohol consumption, drinking behaviors, and alcohol-related problems. Hazardous use cutoff defined as 10 or above.
Drug Use Disorders Identification Test (DUDIT) [46]	11-item self-administered screening instrument for drug-related problems. Cutoff >2 for females and >6 for males.
Patient Health Questionnaire (PHQ-9) [47]	Measure of depression severity, cutoff score of 10 (moderate depressive symptoms).
Generalized Anxiety Disorder Assessment (GAD-7) [48]	Measure for symptoms of anxiety, cutoff score of 10 (moderate anxiety symptoms).
PTSD Checklist for DSM-5 (PCL-5) [49]	20-item self-report measure that assesses the symptoms of PTSD according to DSM-5. Cutoff score of 31 and above for any Criterion A traumatic event endorsed in Life Events Checklist.
Life Events Checklist for DSM-5 (LEC-5) [50]	Assesses exposure to 17 events known to potentially result in PTSD or distress and includes one additional item assessing any other extraordinarily stressful event not captured in the first 16 items.
Health Questionnaire (females only)	This will ask participants about their menstrual cycle and will be conducted during the consent meeting.
*7 days at-home sleep monitoring*	
Sleep diary	Daily questionnaire asking about nighttime sleep, naps, alcohol and caffeine intake, medication, and flu symptoms. A separate version for those who menstruate queries menstrual cycle.
*Lab session*	
Mind Wandering Questionnaire (MWQ) [51]	5-item scale measuring the frequency of mind wandering.
Mindful Attention Awareness Scale (MAAS) [52]	15-item scale designed to assess a core characteristic of mindfulness.
Maladaptive Daydreaming Scale (MDS) [53]	16-item questionnaire designed to assess maladaptive daydreaming.
Perseverative Thinking Questionnaire (PTQ) [54]	15-item questionnaire assessing repetitive negative thinking (non-disorder specific).
Leuven Obsessional Intrusions Inventory (LOII) [55]	50-item self-report instrument designed to measure the frequency of intrusive thoughts, images, impulses, and doubts.
*Post-lab session: At-home cognitive testing and sleep monitoring*	
Sleep diary	Same as above.
Intrusive memory diary [13]	Diary of intrusive memories. Participants complete at least once per day following the lab session. 6 questions about timing, type, and content of intrusive memories, including level of distress.

### Ethics approval

This project has been approved by the Monash Human Research Ethics Committee (project number 40198) and is conducted at the Monash University Sleep and Circadian Medicine Laboratory, Melbourne, Australia.

During the initial phone call following expression of interest, participants are provided with extensive details about the study. Following this, if participants are interested, they are sent the consent form. The consent form includes information about research procedures, potential risks, reimbursement, confidentiality, and freedom to withdraw from participation. Participants also consent to data being used for future projects. Once the consent form is returned, participants are sent the screening criteria link.

A post-participation debrief meeting is held with each participant to identify any ongoing intrusive memories or distress. All debrief meetings are conducted by the project coordinator (J.O.), who is a clinical psychologist. If necessary, resources are provided to participants for community-based services. Following completion of all study components, participants are reimbursed $200 AUD, prorated if the participant discontinues early.

### Procedure and materials

At-home sleep monitoring. For the duration of the experiment, participants wear actigraphy (GENEActiv, Activinsights Ltd., wrist-worn device) and complete daily sleep diaries. For the first eight nights, participants follow a regular sleep schedule, although they wake 2 h early on the day of the lab session. Participant sleep schedules, wake time on day 0 and the timing of the lab session are determined during an initial meeting (day −7 or the previous day) and is based on their regular sleep schedule. Adherence is documented via the sleep diaries and actigraphy.

#### Lab session (day 0)

##### Questionnaires

Five self-report questionnaires assessing spontaneous cognition are completed during polysomnography set-up at the beginning of the lab session. These include mind wandering, mindful attention, maladaptive daydreaming, perseverative thinking, and obsessional intrusions. Each measure is described in Table 2.

##### Polysomnography

Polysomnography recordings for each 2-h sleep or wake opportunity include a 6-channel electroencephalogram (EEG) montage, using electrodes F3/F4, C3/C4, and O1/O2. Eye movement is monitored through electrooculogram and chin activity through electromyogram. Electrical activity of the heart is also measured via electrocardiogram. To screen for obstructive sleep apnea and periodic limb movement disorder, measurement of pulse oximetry and limb movement is included. Participants assigned to the wake group are also monitored with polysomnography throughout the controlled wake period, except for pulse oximetry. Polysomnography recordings are not scored but are monitored throughout the 2-h wake opportunity. Researchers interact with the participant at the first sign of excessive sleepiness, including: (a) no longer engaging in activities provided; (b) closed eyes with reduced alpha, or (c) presence of low amplitude, mixed-frequency activity.

Primary sleep variables for the lab session nap include total sleep time, REM sleep percent, and duration. Examining REM is based on findings linking REM prior to [56] and post-experimental trauma exposure [26] with fewer intrusive memories and reduced distress associated with intrusions, respectively. Secondary sleep outcomes include slow wave sleep (SWS)% and duration. We will also explore the influence of microstructural alterations to frequency-specific EEG activity (eg, slow wave activity and theta and beta frequency during REM and NREM) during the nap on intrusion frequency and recognition memory task performance using quantitative spectral analysis of EEG. Spectral power analysis of EEG data is based on a previous study which found REM theta predicted fewer re-experiencing symptoms post trauma film exposure [24]. Exploratory analysis may include examining nap fragmentation and spindle analysis of NREM stage 2 sleep based on previous findings implicating higher spindle density in fewer reported intrusions [16].

##### Trauma film paradigm

The trauma film paradigm is used in this study to elicit intrusive memories [17]. Clips from two films are viewed in a small, sound attenuated room, and presented on a 24-inch screen with over-ear headphones for audio. Participants first complete a questionnaire about their current emotional state. They watch the trauma film (~12 min in total), which includes two scenes, one from the film Irréversible [57] and the other from a UK public safety advertisement on the dangers of texting and driving [58]. Both film scenes are fictional and have been used in previous studies [56, 59–63]. Film order is counter-balanced. Whilst participants view the films, we ask they maintain their concentration and continuously look at the scene. Upon completion, they rate their current level of distress and degree to which they were able to pay attention to the films. A camera in the room is used to monitor participants’ attention to the film. Participants are told prior to participating in the study they may watch positive, neutral or negative scenes, and are informed about the contents of the negative scenes prior to watching.

#### Recognition and mind wandering tasks

Following the 2-h sleep or wake opportunity, participants complete two tasks. The first is a recognition task. Participants view targets (images from the films) and lures (similar images never seen) and are required to judge whether they witnessed these during the film. The task includes 64 images in total and takes ~10–15 min to complete. Recognition memory task performance will be assessed with the discriminability index (*d-prime* = transformed hits rate − transformed false alarm rate). As a planned secondary analysis, performance on targets, and lures will be individually examined as findings in the literature are inconsistent as to whether performance on targets or lures is driving sleep-related effects on *d-prime* [13, 40].

Mind wandering or off-task thought is related to failures of attention on a range of different sustained attention tasks [64]. The mind wandering task in the current study is an adapted version of a sustained attention to response Go/NoGo task, which has previously been used to elicit and examine mind wandering [65]. Participants are instructed to pay attention to a series of digits and press a button for all digits except the digit 3. The presentation of stimuli is stopped at random times (every 30–70 s) to probe mental state (eg, on/off task) and vigilance of the participants with a series of questions, taking ~18–20 min to complete. The primary outcome from the mind wandering task is frequency of mind wandering.

##### Intrusive memory diary (days 0–5)

Following completion of the cognitive tasks, participants are provided with a definition of intrusive memories and instructions for completing the intrusive memory diary. The diary is completed online, accessible via smartphone, or computer. There are a total of seven questions, including the date and time of the intrusion, the type (eg, image, sound, thought(s)), content of the intrusive memory, whether the intrusive memory was triggered, and level of distress and vividness rating. This diary was based on an online diary used in a trauma film study by Zeng et al. (2021) [13].

An email with the intrusive memory diary link is sent to participants as soon as they leave the lab on day 0. Participants are instructed to complete the diary as soon as possible following the experience of an intrusive memory, completing this as many times as needed. A reminder with the intrusive memory diary link is also sent via text message to participants each evening (between 1 and 3 h prior to scheduled bedtime, based on the previous week), beginning the first evening following the lab session. Participants are instructed to fill out the diary at the end of the evening after receiving the text reminder, even if they have not experienced an intrusive memory. Participants can select an N/A option to each of the diary questions, reducing demand characteristics to report an intrusive memory even if one did not occur. Thus, participants complete the diary a minimum of six times. The primary outcomes from the intrusive memory diary are the total number of intrusive memories following the lab session and number of intrusive memories each day. Secondary outcomes include examining level of distress ratings (1 = not at all distressed, 9 = extremely distressed) when intrusive memories occur.

##### Post-lab session sleep monitoring (days 0–5)

Sleep (at-home) for five nights following viewing the trauma film is measured via actigraphy and sleep diaries. The night following the lab session, participants are required to follow their assigned sleep schedule. Across the remaining four nights participants are not required to follow a regular sleep schedule on these nights. Primary sleep variables measured during this time will be objective total sleep time and sleep efficiency. Secondary variables will include sleep diary-based variables (subjective total sleep time and sleep efficiency).

##### Post-lab session tasks (days 1–5)

**Figure 2:**
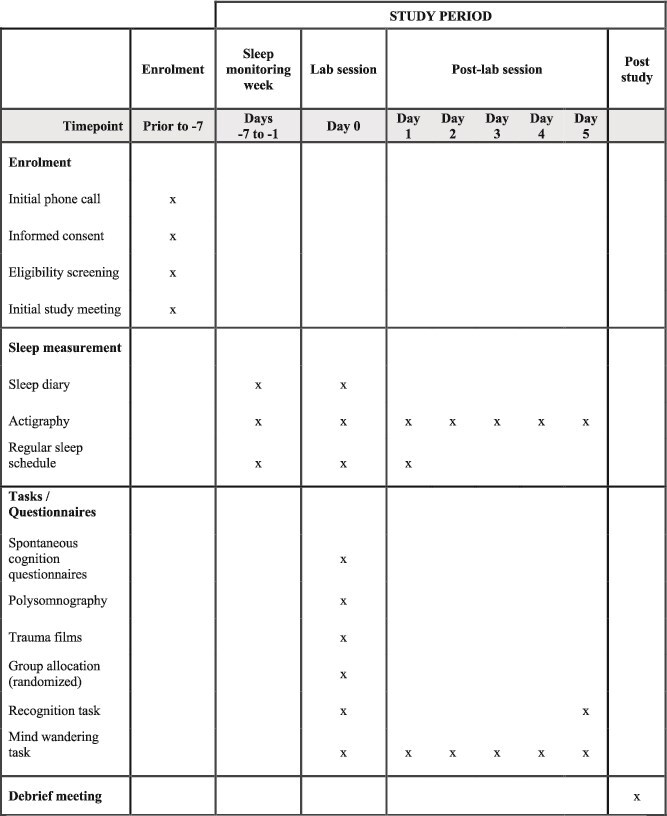
Schedule of enrolment, study questionnaires, and tasks.

For the 5 days following the lab session, participants complete daily intrusive memory diaries and daily mind-wandering task. Participants are instructed to complete the mind wandering task between the hours of 14:00 and 17:00 each day in a quiet environment. Participants are reminded via text and email each day at 13:55 to complete this task. They are required to complete this task on a computer or laptop with a hard keyboard. The recognition task is repeated once on day 5, with an instruction to complete this in a dark room or after sunset to ensure they can clearly view the images. Participants are instructed to complete the recognition task on a computer screen (rather than a smartphone) and are reminded via text and email on day 5. The links to the recognition task on day 5 and the mind wandering task each day are included in the reminder email. Figure 2 details the schedule of enrolment, study questionnaires, and tasks.

### Adherence and monitoring

Adherence to the sleep schedule and task completion is monitored throughout participation.

#### Sleep schedule adherence

The sleep schedule is set in collaboration with the participant based on self-reported habitual sleep schedule. Each night and morning of the study, participants call the study phone and leave a message to indicate sleep and wake times. Sleep diaries and participant phone calls are monitored daily by the researchers. Within the first three nights of participation, if it is clear that the participant’s sleep schedule is different than what was initially set, the sleep schedule is adjusted to match the participant’s actual sleep schedule, as long as it stills falls within the study criteria (they do not sleep before 22:00 or after 01:00 and do not wake before 06:00 or after 09:00). If the sleep schedule is adjusted, participant wake time on the day of the lab session is also adjusted (to 2 h prior to regular wake time), as is lab session timing. Based on sleep diaries and sleep/wake time phone calls, participants are excluded from the study if they do not adhere to the sleep schedule for at least five nights. Non-adherence includes napping, going to sleep too early, or oversleeping. Participants are also excluded from the study if they wake 30 min after their scheduled wake time on the day of the lab session. If a participant is non-adherent to the sleep schedule, participation is rescheduled if there is agreement that they will be able to adhere for the rescheduled week. After the lab session, the sleep schedule is followed for one more evening. If participants are non-adherent to the sleep schedule on this evening (eg, napping following the lab session), they are excluded from the study and cannot be rescheduled.

#### Trauma films and intrusive memory diary adherence

Participants must be observed to be looking at the screen at least 80 per cent of the time when watching the films or to have rated their level of attention to the most graphic parts of the film as at least 5, on a 1 (*none at all*) to 9 (*total attention*) point scale. Participants are asked to complete the intrusive memory diary online each day and are contacted the following day via email if they have not completed it.

#### Mind wandering task adherence

Participants are contacted during the study if they do not complete tasks or are non-adherent. Participants who do not complete the mind wandering task between the hours of 14:00 and 17:00 are contacted by researchers on the day via email and asked to complete the task as soon as possible. If the participant cannot complete the task prior to 12 h after their wake time for that day (based on the sleep diary and phone call), they are asked not to complete the mind wandering task for that day.

## Analytic strategies

All analyses will be conducted in R.

Aim 1: The effect of a daytime nap (vs wake) on total intrusive memory frequency will be analyzed with a generalized linear mixed-effects model. Sex and day will be included as covariates. A random intercept for participant will be included to account for repeated measures across days.

Aim 2: The effect of sleep-related memory consolidation on intrusive memory frequency will be analyzed with regression and mediation analysis. Specifically, Hypothesis 2a (a nap will lead to greater recognition memory for the trauma film) will be examined with multiple regression, whereby recognition memory immediately after the nap/wake will be predicted by Condition. For Hypothesis 2b (recognition memory will mediate the relationship between sleep and intrusive memories), we will utilize mediation analysis, with Condition as the explanatory variable, recognition memory immediately after the nap/wake as the mediator, and total number of reported intrusive memories as the dependent variable. A Monte Carlo power analysis was conducted for the mediation model. A sample size of *n* = 114 has limited power to detect the indirect effect. Nonetheless, given the strong theoretical rationale for the mediation analysis and the value for future research in determining effect sizes, we will conduct the analysis and consider Hypothesis 2b as a secondary analysis. We will report effect sizes and confidence intervals of each path. We will also conduct parallel exploratory analyses for both Hypotheses 2a and 2b, utilizing recognition memory on day 5, rather than memory immediately following the nap/wake. This will explore the role of longer-delay memory consolidation on intrusive memory. Note, Hypothesis 2b may appear dependent on the success of Aim 1.

Aim 3: The effect of overnight sleep on next-day mind wandering and intrusive memory frequency will be analyzed with mixed models. In two separate analyses, Hypothesis 3a (reduced sleep will be associated with increased mind wandering and increased intrusive memories the next day) will use sleep (total sleep time, sleep efficiency) each night to predict frequency of mind wandering and number of intrusions the next day. Hypothesis 3b (mind wandering will mediate the relationship between sleep and intrusive memories the next day) will be tested with two mediation analyses. Sleep duration each night will serve as the explanatory variable, mind wandering the next day will serve as the mediator, and number of intrusions the next day will be the dependent variable. Hypothesis 3b is also considered a secondary analysis. We were unable to perform a power analysis for this mediation as there are no previous studies examining the effect of sleep on both spontaneous cognition and intrusive memories, and thus, no basis for estimating effects sizes. If this mediation model is significant, as an exploratory analysis, we will also run a moderated mediation to explore if Condition moderates the relationship between mind wandering and intrusive memories, in the context of the mediation analysis. A separate set of models with sleep efficiency as the explanatory variable will also be run as an exploratory analysis. If the structure of the data (eg, excessive zero counts in the intrusive memory data) does not allow for mediation, alternative analytical strategies will be developed to address the same aim for Hypotheses 2b and 3b.

For Hypothesis 1 and Hypothesis 3a, we will run additional secondary analyses to examine the influence of trait mind wandering, mindful attention, frequency of intrusive thoughts, perseverative thinking and maladaptive daydreaming on the relationship between sleep and intrusive memories. Each trait measure will be examined in a separate model, where the given trait measure is added to the primary model for Hypotheses 1 or 3a. We will examine the main effect of the trait measure, as well as the interaction between the trait measures and the primary effect of interest for each model. These analyses will allow us to determine if trait factors theoretically related to the vulnerability to intrusive memory or mind wandering influence the sleep effect.

### Data exclusion

All participants who complete the study will be included in the analysis, except as specified below. Data will not be imputed for the primary outcomes in this study. We will use sufficiently robust statistical models that accommodate missing data. Prior to the lab testing session (day 0), participants will be excluded if they have not adhered to at least five nights of the assigned sleep schedule and/or not adhered to the assigned sleep schedule for the two nights prior to the testing session. Participants are excluded from the study if they wake 30 min after their scheduled wake time on day 0. Participants are also excluded from the study if they do not follow the sleep schedule (or nap) following the lab session on day 0. For questionnaires assessing spontaneous cognition completed during PSG set-up on day 0, we may impute data for any missing responses using multiple imputation.

For the mind wandering task, for any given aim, participants will be included if they provide at least 1 day’s worth of data for that aim. We will exclude data where the participant has not understood the task or is not a conscientious responder (operationalized as below 50 per cent performance on both Go and NoGo trials). Participant understanding of the task will also be checked during a debrief at the end of the study. Timing of the mind wandering task is checked against sleep diary data, participant phone calls (regarding sleep and wake timing) and actigraphy data. If the task is completed more than 12 h post-wake time, data for that day will be excluded.

If participants report that they did not pay sufficient attention to the most graphic sections of either film and they are not observed to be watching the screen at least 80 per cent of the time, their data for the whole duration of the study will be excluded. For the intrusive memory diary, data will not be imputed. Only intrusive memories of the films are included. Unclear diary entries are discussed with participants during the debrief meeting at the end of the study. If the participant does not complete the intrusive memory diary for the entire duration of the study, their data will be excluded. We will not exclude participants who report a high number of intrusions, instead winzorization will be performed for outliers.

We will exclude data from the recognition memory task if the participant is not a conscientious responder. This is operationalized as below 50 per cent performance for both targets (images they have seen) and lures (images they have not seen).

### Exploratory analyses

Several exploratory analyses will also be conducted in this study. Specific examples include: (1) The influence of sex on intrusive memory frequency will be examined, as some evidence suggests females experience more frequent intrusive memories following actual [66] and analog [67] trauma exposure during certain phases of the menstrual cycle. (2) In the day-to-day analysis, we will analyze the effect of accumulation of sleep over multiple days on mind wandering and intrusive memory frequency. (3) We will further expand the temporal aspects of the model in Hypothesis 3 to include sleep the night before the lab session (night −1, day 0), to examine sleep’s effect on mind wandering and intrusive memories over a 6-day period. (4) The effect of the nap on mind wandering and intrusive memories on day 0 will also be examined. (5) A further outcome of the mind wandering task will be how many reports of mind wandering are related to the film.

### Subgroup analysis

Exploratory subgroup analyses may include examining only participants who experience intrusions, excluding those who do not experience any intrusive memories following the trauma film. We may also examine intrusion frequency for only those participants who experience a level of distress of four and above (out of 9) when watching the trauma films (completed immediately following the films).

### Data management plan

All sleep recordings including polysomnography and actigraphy recordings are stored on a Monash University server. Self-report data, including sleep diaries and intrusive memory diaries, are collected using Qualtrics. Links are sent to participants via text reminder. Questionnaires assessing trait-level spontaneous cognition are collected via Qualtrics or pen-and-paper during PSG set-up on the day of the lab session. The recognition memory task is also completed using Qualtrics. The mind wandering task is completed using Inquisit (Millisecond Software). Participants are provided with a participant code prior to completing any measures, including screening questionnaires. Participant codes are associated with questionnaires, diaries/tasks, and polysomnography data. Only researchers associated with the study have access to Qualtrics and the Monash University server.

### Data sharing and dissemination

Final data analysis will begin after completion of data collection. Partial study results may be presented at national and international conferences. Full study results will be disseminated via peer-reviewed publication and conference presentations. Following publication, a summary of results will be sent to participants who completed the study, in recognition of their participation. Following publication of primary outcomes, de-identified participant data and code will be made available in an accessible data repository.

## Discussion

A growing body of research demonstrates sleep reduces the frequency of intrusive memories following experimental trauma exposure [13–16, 23, 24]. Studies examining trauma-exposed populations and persons with PTSD also suggest sleep may affect the frequency of intrusive memories both in the early aftermath [10, 11] and years after trauma exposure [12]. The current study builds on previous research examining the effect of sleep on intrusive memories following experimental trauma exposure, to examine *how* sleep exerts this effect on intrusive memory frequency. To do this, we are experimentally manipulating sleep immediately following viewing a trauma film and monitoring sleep over the following five nights. Participants also record intrusive memories, report mind wandering frequency during a sustained attention task and complete a recognition task for trauma film stimuli to examine whether memory consolidation and executive control over spontaneous cognition mediate the relationship between sleep and intrusive memories.

While the trauma film paradigm is a well-established method to elicit and study intrusive memories, there are several limitations to be considered. The trauma film is not real trauma exposure and distress in response to the film is unlikely to be as high as in real world trauma. Despite this, the paradigm consistently produces a sufficient analog trauma response to be modulated by sleep, although distress associated with intrusive memories in this paradigm is not reliably elicited [13, 14, 26, 40, 68]. Other external validity issues include the peripheral experiences related to the primary trauma incident, which may further increase distress (eg, attending an emergency department or providing a statement to police), which this paradigm is unable to simulate. As participants are informed that they may watch a disturbing film, there may also be a risk of selection bias. Those choosing not to participate may be more concerned about potential film content, and possibly more likely to experience the intrusive memories we are attempting to measure.

During development of the recognition task, pilot participants rated images for valence, distress, and clarity, to ensure these domains were similar across targets and lures (see Supplementary Material S1). As this was a separate cohort of participants who were not exposed to the trauma films, there is still likely to be inter-individual variability across valence and distress associated with the images, which could affect recognition. However, we did not include distress ratings of each image during the recognition task, to reduce participant burden. Finally, all tasks (mind wandering and recognition) in the final 5 days of the study are completed at home. While participants are instructed to complete the tasks in quiet environments and task timing and performance is monitored by researchers; these are completed in less controlled environments relative to the lab session on day 0. At the same time, task completion from home reduces participant burden in this study, enabling the investigation of a potentially important mechanism (executive control over spontaneous cognition) that may have otherwise been difficult to examine over a 5-day period in laboratory conditions.

Despite these limitations associated with the use of trauma films, this paradigm does reliably induce intrusive memories of the films and has been extensively used to examine factors influencing intrusive memory frequency [17]. As prior research has examined sleep and intrusive memories in both healthy and trauma-exposed populations, a strength of the present study lies in its aim to replicate previous findings and extend this line of research in several ways. Firstly, we are extending upon three studies which have examined explicit trauma memory following a period of overnight sleep [13, 23, 40] by examining this following a nap. If naps enhance explicit trauma memory in addition to reducing intrusive memories, this will provide further support for the memory consolidation account [36]. Secondly, to investigate an executive control hypothesis, we are examining day-to-day relationships between sleep and intrusive memories, similar to research examining multiple periods of sleep in the immediate aftermath [10, 11] and many years following actual trauma exposure [12]. Finally, we are investigating the effect of sleep architecture and micro architecture on intrusive memory frequency, following studies linking REM to reduced intrusion-related distress [26] and sleep micro-architectural features (N2 sleep spindles, REM theta) to fewer intrusions [16, 24]. Moreover, persons with PTSD exhibit REM abnormalities in addition to sleep disturbance [69, 70]. Overall, findings from this study will provide insights into how sleep affects the development and possible maintenance of a key re-experiencing symptom of PTSD and provide further direction for mechanistic research, including examining these mechanisms in trauma exposed populations and/or those experiencing sleep disorders.

## Supplementary Material

Supplemental_Document_Pilot_Study_Description_zpag022
