# Supplementary material for: Mechanisms through which sleep influences intrusive memories: protocol for a trauma film paradigm study
Source: Sleep Adv. 2026 Feb 14;7(1):zpag022. doi: 10.1093/sleepadvances/zpag022 (PMC13174388; doi:10.1093/sleepadvances/zpag022)
Supplement: Supplemental_Document_Pilot_Study_Description_zpag022 [file supplemental_document_pilot_study_description_zpag022.pdf]

Mechanisms through which sleep influences intrusive memories. Protocol for a trauma film paradigm study.

Jessica Ogden[1,2], Laura Jobson[1,2], Sean P. A. Drummond[1,2]

1 School of Psychological Sciences, Monash University, Clayton, Victoria, Australia,

2 Turner Institute for Brain and Mental Health, Monash University, Clayton, Victoria, Australia

Correspondence to: Jessica Ogden, Monash Sleep and Circadian Rhythms Program, Monash University, 1/270 Ferntree Gully Rd, Notting Hill 3168, Victoria, Australia.

Email: jessica.ogden@monash.edu

Mechanisms through which sleep influences intrusive memories. Protocol for a trauma film paradigm study.

Pilot study description. Two pilot studies were conducted to: 1) ensure the trauma films induced intrusive memory frequency comparable to previous research; 2) develop the recognition task to examine hypothesis two; and 3) pilot the recognition task and a shortened version of the mind wandering task (adapted from Andrillon et al., 2021) [65] to examine hypotheses two and three. Exclusion criteria for both pilots were the same as the main study except for excluding those who were French speakers for pilot 1, as these participants did not view the trauma films.

The aim of the first pilot study was to develop the recognition task for hypothesis 2. Using Prolific, an online participant recruitment platform, participants (n=49) from Australia, the USA, New Zealand and Canada rated 86 images including 43 targets (images from the films) and 43 lures (images from other parts of the films or other films/images). Participants rated the 86 images on valence ('how negative or positive is this image?'), distress ('how distressing is this image?') and clarity ('how easily can you discern the objects in this image?'), each on a 7-point scale. They were paid $11.50 AUD per hour to complete the screening questions and ratings (15-20 mins).

The aim of pilot study 2 was to finalise the trauma films and recognition and mind wandering tasks. Pilot 2 excluded the sleep manipulation and ran for six days. Participants (n=11) attended the lab, viewed the trauma film, and completed the recognition and mind wandering tasks after a 1.5-hour delay period. They then completed an intrusive memory diary and mind wandering task for five days from home (see Figure 1 for protocol). This delay period was comparable to the wake group in the main study, except for participants being able to complete university work or read a book during this time. The mind wandering

task was originally shortened to 10-12 minutes in length to reduce participant burden for the main study, given other at-home demands in the study (sleep and intrusive memory diaries, phone calls regarding sleep/wake timing and delayed recognition task). The delayed recognition task was completed at home on day 6 (5-day delay as in the main study). Participants were reimbursed $75 AUD for pilot 2.

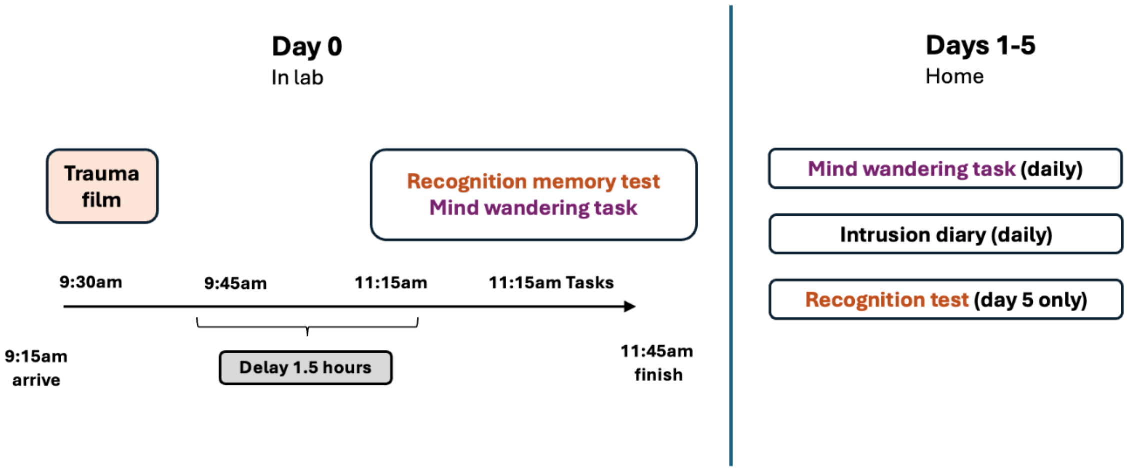

Figure 1. Pilot 2 protocol. Participants arrived at the lab at 9:15am, watched the trauma films at 9:30am, followed by a 1.5-hour delay. The recognition and mind wandering tasks were completed following the delay period. Participants then completed the mind wandering task from home each day between 15:00-17:00 and the recognition task on the final day. Participants were also instructed to complete the recognition task in the evening (after sunset) or in a dark environment, as in the main study.

Intrusive memories. Participants reported a mean of 3.8 (range 0-20) intrusive memories, comparable to previous studies. For example Gvozdanovic et al. (2023) [14] reported 4.19 (*SD* = 0.8) as the mean frequency of intrusions for the wake group, compared to a nap; Zeng et al. (2021) [13] reported a mean of 1.57 (*SD* = 1.68) intrusive memories for the overnight wake group compared to overnight sleep and Kleim et al. (2016) [16] reported a mean of 5.91 (*SD* = 3.46) intrusions for a wake group shown the trauma film in the morning, compared to the sleep group with evening trauma film exposure. There was a large standard

deviation (5.6) in this pilot, which was expected given the small sample size. However, this is also representative of studies examining sleep and intrusive memory frequency.

Recognition task. Based on ratings from pilot 1 and performance on this task in pilot 2, the task was reduced to 64 images (32 targets / 32 lures) to be used in the main study. Images were removed based on extreme ratings (i.e., images rated as extremely positive or negative or images that were too unclear) from pilot 1. Performance on the recognition task during pilot 2 was used to further remove images with more extreme scores (i.e., every participant either correctly or incorrectly identifying the image as old/new). T-tests were used to compare ratings of valence, distress, and clarity ratings across targets and lures. With the final 64 images, valence, distress and clarity of image ratings were not significantly different across targets and lures.

Mind wandering task. Frequency of mind wandering reported by participants over the 10-minute task (across 9 mind wandering probes) was 18%. This did not reflect the previous study using this task to measure mind wandering where participants reported mind wandering around 38% of the time [65]. Therefore, the task was increased from 10-12 mins to the original 18-20 mins in length (18 mind wandering probes). For the main study, during the 5 days of task completion at home, the period in which participants are reminded to complete the task was extended by 1 hour (14:00-17:00 in the main study), roughly 8 hours after wake, following the inclusion of participants who tend to wake between the hours of 6:00 – 9:00.
